# The Heart’s Hidden Neural Network: Interplay Between Intracardiac Ganglia, Fibrosis and Cardiac Remodeling

**DOI:** 10.3390/ijms27031582

**Published:** 2026-02-05

**Authors:** Jacques-Antoine Gemayel, Aurelien Chatelier, Patrick Bois, Nassim Fares

**Affiliations:** 1 Laboratory of Research in Physiology and Pathophysiology, Faculty of Medicine, Saint Joseph University of Beirut, Beirut 1104 2020, Lebanon; jacques-antoine.gemayel@net.usj.edu.lb; 2 PReTI Laboratory, UR 24184, University of Poitiers, 86000 Poitiers, France; aurelien.chatelier@univ-poitiers.fr (A.C.); patrick.bois@univ-poitiers.fr (P.B.); 3 CERVO Brain Research Centre, Faculty of Medicine, Université Laval, Québec City, QC G1J 2G3, Canada

**Keywords:** intrinsic cardiac nervous system, intracardiac ganglia, cardiac neurons, neuroinflammation, neurofibrosis, IL-6/STAT3, TGF-β signaling, PI3K/AKT, autonomic remodeling, arrhythmogenesis, neuromodulation, diabetic cardiac remodeling

## Abstract

The heart’s performance relies on its contractile and rhythmic properties, which are modulated not only by extrinsic autonomic inputs but also by the intrinsic cardiac nervous system (ICNS), a distributed network of intracardiac ganglia and neurons that integrates local sensory, autonomic, and inflammatory signals. Growing evidence indicates that cardiac fibrosis and neuronal remodeling are intertwined processes within this network. This review synthesizes current knowledge on molecular, structural, and functional remodeling of the ICNS to drive neurofibrosis, autonomic imbalance, and arrhythmogenesis. We outline ICNS anatomy and neurochemical diversity, then summarize core fibrotic mechanisms, fibroblast activation, extracellular matrix dynamics, and inflammatory signaling, and map these onto intracardiac ganglia. Across diabetes, myocardial infarction, heart failure, and neuroinflammatory states, shared pathways (e.g., IL-6/STAT3, TGF-β/SMAD, PI3K/AKT, MAPK/ERK, oxidative stress) suppress neuronal excitability, promote neuron–glia–fibroblast coupling, and culminate in neurofibrotic remodeling. We integrate functional data linking these changes to autonomic dysregulation and arrhythmia vulnerability. Future priorities involve constructing detailed human ICNS atlases and applying single-cell and spatial multi-omics to better characterize intracardiac neurons, their circuitry, and their interactions with fibroblasts and immune cells. These insights will be essential to inform targeted neuromodulation and anti-fibrotic interventions. The ICNS is a dynamic regulatory hub whose cells and circuits participate directly in cardiac fibrosis and electrical instability. Recognizing neurofibrosis as a companion process to myocardial fibrosis reframes therapeutic strategy toward preserving both neural and myocardial integrity.

## 1. Introduction

The heart is often described as a mechanical pump, but its function depends on far more than muscle and valves [1]. Healthy cardiac performance relies on a delicate balance between the organ’s own intrinsic properties and the regulatory systems that shape how it responds to physiological demands [2]. One key element in this balance is the way the heart remodels its structure over time [3]. When this process becomes dysregulated, fibrosis develops, an excessive buildup of extracellular matrix that stiffens the myocardium, disrupts electrical conduction, and contributes to a wide range of cardiac diseases [4]. Traditionally, fibrosis has been viewed primarily through the lens of fibroblast biology.

However, recent findings suggest that this view is incomplete. Across multiple organ systems, neurons interact closely with fibroblasts and can directly influence fibrotic remodeling [5]. Through neurotransmitter release, paracrine signaling, and structural communication, neurons help shape how tissues respond to injury, inflammation, and metabolic stress [6]. This broader perspective raises an important question: if neurons elsewhere in the body modulate fibrosis, could similar neuro-fibrotic interactions also exist within the heart?

Addressing that question requires looking more closely at the heart’s own nervous system. The intrinsic cardiac nervous system (ICNS), or what we call intracardiac ganglia (ICG) neurons, a distributed network of ganglia, sensory neurons, local circuit neurons, glia, and interconnecting fibers, sits directly within the myocardium and plays a critical role in fine-tuning cardiac rhythm and contractility [7,8]. Although the ICNS has long been recognized as essential for beat-to-beat regulation, its involvement in structural cardiac remodeling has only recently become a topic of interest [9].

We suggest that cardiac neurons and fibroblasts do not operate as isolated cell types. Instead, they participate in a shared microenvironment where neural activity, inflammatory signals, and extracellular matrix dynamics continuously influence one another [10]. Work from non-cardiac tissues has shown that neurons can drive or restrain fibroblast activation, alter extracellular matrix composition, and shape the trajectory of tissue repair [11,12,13]. These insights have opened the door to the idea of a “neuro-fibrotic axis” in the heart, where neuronal–fibroblast crosstalk within the ICNS may contribute to both adaptive and pathological remodeling.

Understanding this interplay is especially important given that fibrosis and neuronal remodeling often occur together in cardiac disease, yet the mechanisms linking these processes remain poorly defined. Clarifying how ICNS neurons, glia, and fibroblasts communicate could reveal new therapeutic targets, not only to reduce fibrosis, but also to preserve cardiac autonomic regulation and prevent arrhythmias.

The purpose of this review is to bring together emerging evidence on how the ICNS is remodeled under physiological and pathological conditions, with a particular focus on the molecular and cellular mechanisms that connect neuronal signaling to fibrotic responses. We synthesize findings from animal models and human studies to highlight how metabolic stress, inflammation, neuroimmune pathways, and extracellular matrix remodeling converge on neuronal and fibroblast networks within cardiac ganglia. Finally, we identify key knowledge gaps and outline future directions for establishing the neuro-fibrotic axis of the ICNS as a meaningful contributor to cardiovascular disease and a promising therapeutic frontier.

## 2. The Intrinsic Cardiac Nervous System and Cardiac Neurons

Despite their central role in autonomic cardiac regulation, the study of cardiac neurons (CN) has been historically constrained by technical limitations and an emphasis on extrinsic autonomic pathways [14]. Unlike the extensively characterized enteric nervous system, the ICNS has received comparatively little attention, resulting in significant gaps in understanding its structure, function, and contribution to cardiac disease [7].

Cardiac neurons within the ICNS are uniquely positioned to integrate sympathetic, parasympathetic, and local cardiac inputs, functioning as a distributed processing network rather than a simple relay for extrinsic commands [15]. Through dynamic modulation of nodal activity, conduction, and contractility, ICNS neurons contribute directly to the beat-to-beat regulation of cardiac performance [16]. The cholinergic components of this network are particularly vital for maintaining ventricular electrophysiological stability and protecting against arrhythmogenic perturbations [17].

Emerging clinical and experimental data indicate that disruption of this delicate autonomic balance, for instance, following atrial fibrillation (AF) ablation or chronic metabolic stress, impairs parasympathetic modulation and promotes heterogeneous sympathetic dominance [18,19]. Such neural dysregulation may create a proarrhythmic substrate even in structurally normal hearts, highlighting the importance of preserving neural integrity within the ICNS [20]. Moreover, in patients undergoing coronary artery bypass grafting, for example, intraoperative botulinum toxin injection into epicardial fat pads significantly reduced the incidence of atrial fibrillation over three years. BTX suppresses autonomic hyperactivity and prevents electrical remodeling, illustrating the therapeutic potential of targeted neuromodulation in arrhythmia prevention [21]. Translating these insights into therapies remains challenging due to limited accessibility of the ICNS, the complexity of targeted neuromodulation, and the current lack of human-specific molecular atlases. Complementary analysis of human ganglionated plexus neurons revealed enhanced excitability and adrenergic shift in atrial fibrillation, with increased synaptic complexity and prolonged firing, implicating neuronal hyperexcitability in arrhythmogenic substrate formation [22]. In addition, premature ventricular contractions also drive arrhythmogenic remodeling by destabilizing neuronal firing patterns within the intrinsic cardiac nervous system. Variable premature ventricular contractions coupling intervals increase repolarization dispersion and disrupt sympatho-vagal balance, revealing the ICNS as both mediator and therapeutic target for neuro-cardiac reflex-driven instability [23].

Understanding the organization and plasticity of cardiac neurons is therefore essential for elucidating the mechanisms underlying cardiac dysfunction and for identifying novel neuromodulatory therapeutic targets.

Anatomically, the ICNS forms a highly organized network of ganglia and interconnecting plexuses embedded within the heart, predominantly along the epicardial surfaces of the atria and ventricles [7]. These ganglia are concentrated within epicardial fat pads, where they form ganglionated plexuses that contain the cell bodies of afferent, efferent, and local circuit neurons [24]. The most densely populated regions occur along the posterior atrial surfaces, particularly around the pulmonary veins and atrioventricular junctions. Distinct ganglia, such as the superior and inferior paraseptal ganglionated plexuses, exert specialized control over the sinoatrial and atrioventricular nodes, respectively, enabling precise modulation of cardiac rhythm and conduction [8].

**Figure 1:**
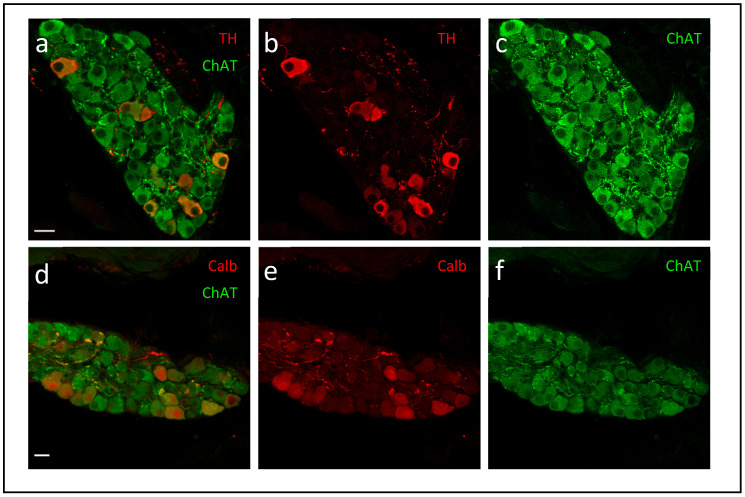
Neurochemical phenotype of intracardiac neurons. Confocal micrographs of mouse intrinsic cardiac ganglia immunostained for Tyrosine Hydroxylase (TH) and Cholin Acetyltransferase (ChAT) (**a**–**c**) or calbindin (Calb) and ChAT (**d**–**f**). All images are projected confocal Z-stacks from sectioned tissue. Scale bars: 20 µm. Images adapted from Lizot et al., 2022 [30].

Histological and neurochemical analyses have revealed that the ICNS contains a diverse population of neurons, including cholinergic, adrenergic, and peptidergic subtypes, underscoring its integrative role in cardiac autonomic control [25,26,27,28]. Most intrinsic cardiac neurons exhibit a predominantly cholinergic phenotype, expressing choline acetyltransferase (ChAT) like Figure 1 shows, the choline transporter (CHT), and the vesicular acetylcholine transporter (VAChT), all of which mediate acetylcholine-dependent parasympathetic signaling [24,26]. This hybrid cholinergic–adrenergic phenotype may allow these neurons to fine-tune local neurotransmission or modulate cardiac excitability through autocrine and paracrine mechanisms, particularly during stress or ischemia [29].

Comparative studies of ICNS across species reveal distinct electrophysiological and structural specializations. Human ICNS exhibit higher excitability, greater after-hyperpolarization, and richer synaptic integration compared with murine and porcine counterparts, emphasizing the translational need for human-based models in cardiac neurobiology [31]. Additionally, Three-dimensional imaging and molecular mapping of the ICNS have further defined its neurochemical heterogeneity, identifying cholinergic, catecholaminergic, and peptidergic subpopulations with diverse firing properties that integrate cardiac control across neuronal networks [25,27,30,32,33].

Moreover, intrinsic cardiac neurons express TrkA and p75 neurotrophin receptors, indicating responsiveness to nerve growth factor (NGF) and a capacity for neurochemical and structural plasticity [34]. This adaptability is especially relevant under pathological conditions such as heart failure or diabetes that lead to neuronal hypertrophy and remodeling. Within the cardiac ganglia, cholinergic and adrenergic fibers are often closely apposed, providing an anatomical substrate for cross-talk that refines the balance between sympathetic and parasympathetic influences [35].

Functionally, the ICNS operates in close coordination with the extrinsic autonomic nervous system, integrating descending inputs from both sympathetic and parasympathetic preganglionic fibers. Preganglionic vagal fibers synapse within the cardiac ganglia, while sympathetic inputs from the stellate and middle cervical ganglia converge upon the same network, establishing a bidirectional communication system [36]. Whereas the extrinsic autonomic system provides broad, systemic modulation of heart function, the ICNS acts locally, refining and contextualizing these signals according to regional myocardial conditions such as ischemia, stretch, or inflammation [7].

Collectively, the ICNS serves as a decentralized regulatory hub, translating global autonomic commands and local sensory feedback into fine-tuned adjustments of cardiac output [37]. Its distributed architecture, neurochemical diversity, and intrinsic processing capacity underscore its role as the heart’s “little brain” [38].

As new molecular and imaging tools begin to map this system with unprecedented precision, the ICNS is emerging as a critical player in cardiac health and disease, one that may hold the key to understanding arrhythmogenesis, neurofibrosis, and autonomic remodeling.

Given this intrinsic network’s susceptibility to metabolic and inflammatory stressors, it is increasingly evident that the same molecular cues driving myocardial fibrosis, particularly TGF-β, IL-6/STAT3, and PI3K/AKT signaling, also reshape neuronal architecture within the ICNS. The following section examines the pathogenesis of cardiac fibrosis (CF) as a foundation for understanding its neural counterpart.

## 3. Pathogenesis of Cardiac Fibrosis

CF is a pathological process characterized by the excessive accumulation of ECM components such as collagen and fibronectin within myocardial tissue. Fibrotic remodeling leads to increased myocardial stiffness and reduced compliance, impairing both contraction and relaxation [39]. This maladaptive process contributes directly to heart failure, arrhythmias, and progressive structural cardiomyopathies [40]. The development of CF reflects a complex interplay between fibroblasts, immune cells, endothelial cells, and signaling molecules, all integrated within the myocardial microenvironment and further influenced by the ICNS. Emerging evidence suggests that neuronal fibroblasts within ICG contribute to fibroblast activation, ECM remodeling, and the progression of fibrotic disease.

### 3.1. Cellular and Molecular Mechanisms of Cardiac Fibrosis

#### 3.1.1. Activation of Cardiac Fibroblasts and Myofibroblasts

Cardiac fibroblasts are the principal effector cells in fibrosis, responsible for ECM synthesis and deposition [41]. Under physiological conditions, cardiac fibroblasts maintain tissue architecture; however, in response to injury, mechanical overload, or metabolic stress, cardiac fibroblasts become activated and differentiate into myofibroblasts, specialized, highly contractile cells characterized by the expression of α-smooth muscle actin (α-SMA) [41]. Myofibroblasts exhibit enhanced proliferative capacity and secrete large quantities of collagen, fibronectin, and proteoglycans, contributing to ECM accumulation and myocardial stiffening [42].

Among the various fibrogenic mediators, transforming growth factor-β (TGF-β) represents a key component of the broader fibrogenic signaling network, acting through both canonical and non-canonical pathways that regulate fibroblast activation and myofibroblast differentiation. While TGF-β signaling is central, it operates in concert with other profibrotic mediators and pathways that collectively drive CF [43]. Acting through TGF-β1-3 isoforms and their receptors, it promotes fibroblast proliferation, differentiation, and ECM gene expression. Recent studies highlight crosstalk between TGF-β and the metalloprotease ADAMTS16, which activates latent TGF-β (LAP–TGF-β) by proteolytic cleavage [44]. This interaction forms a feed-forward loop that intensifies fibroblast activation and cardiac hypertrophy. Inhibition of TGF-β signaling, such as with TGF-β neutralizing antibodies (TGF-β–NAb), has been shown to mitigate fibrosis and restore cardiac function in experimental models, underscoring this pathway’s therapeutic relevance [45].

#### 3.1.2. Synthesis and Deposition of the Extracellular Matrix

The cardiac ECM provides both structural integrity and a biochemical framework regulating cell–cell communication [46]. It is primarily composed of fibrillar collagens (types I and III), glycoproteins, proteoglycans, and latent growth factors [39]. During fibrosis, activated fibroblasts produce excessive collagen I and III, altering the collagen I/III ratio, a hallmark of pathological stiffening. Collagen I dominates in hypertensive and ischemic remodeling, producing rigid, inelastic myocardium, whereas collagen III maintains elasticity.

Emerging data also identify collagen V as a regulatory element in ECM organization. In myocardial infarction (MI) animal models, collagen V expression during scar maturation modulates fibroblast mechanotransduction by influencing integrin expression, restraining further activation [47]. Thus, CF is driven not only by collagen production but also by ECM structural dynamics that perpetuate mechanical and biochemical stress signaling.

#### 3.1.3. Inflammatory Signaling and Immune Response

**Figure 2:**
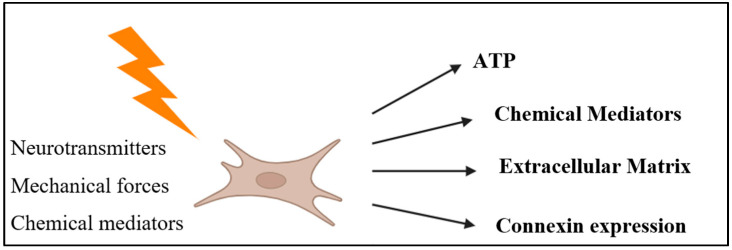
Neurochemical and mechanical activation of cardiac fibroblasts leading to paracrine and extracellular responses. Cardiac fibroblasts respond to neurotransmitters, mechanical forces, and chemical mediators by releasing ATP, chemical mediators, and components of the ECM, while also modulating connexin expression. These outputs contribute to fibroblast–neuron and fibroblast–fibroblast communication within the cardiac microenvironment.

Inflammation acts as both a trigger and amplifier of fibrosis. Elevated myocardial levels of TNF-α, IL-1β, and IL-6 correlate with collagen deposition and matrix metalloproteinase (MMP) activation in human and experimental cardiomyopathies [48]. These cytokines recruit macrophages and promote fibroblast-to-myofibroblast transition, establishing a self-reinforcing inflammatory-fibrotic loop. The specific profile of signaling factors released by cardiac fibroblasts is highly dependent on external stimuli, which may include chemical cues (e.g., growth hormones, pro- and anti-inflammatory cytokines), mechanical stress, electrical signals, or hypoxic conditions [49]. As illustrated in Figure 2, cardiac fibroblasts respond to various neurochemical, mechanical, and paracrine stimuli by releasing a wide array of molecules, including ATP, chemical mediators, ECM components, and connexins, that collectively contribute to fibrosis, inflammation, and cardiac remodeling.

Recent findings identify interleukin-27 (IL-27), produced mainly by macrophages and dendritic cells, as a potent profibrotic cytokine. After MI, IL-27 upregulation in F4/80^+^ macrophages promotes fibroblast differentiation and collagen synthesis, linking innate immunity to fibrotic progression [48,49]. Moreover, IL-6 and TGF-β signaling in fibroblasts drives α-SMA stress fiber formation and matrix contraction, with IL-6 transitioning from a proinflammatory to profibrotic mediator as the disease progresses [4,50,51]. This duality illustrates how immune–fibroblast crosstalk dictates both the initiation and persistence of CF [52].

#### 3.1.4. Tissue Repair and Remodeling

While fibrosis is often associated with pathology, fibroblast activation is essential for post-infarction repair. Following MI, fibroblasts proliferate in the border zone to stabilize necrotic myocardium and prevent rupture [53]. However, persistent activation transforms this reparative response into maladaptive remodeling [54].

Although CF has traditionally been viewed as a process confined to the myocardium, mounting evidence indicates that similar molecular mechanisms operate within the intrinsic cardiac ganglia. Fibroblast activation, cytokine signaling (particularly IL-6/STAT3 and TGF-β), and oxidative stress also drive neurofibrotic remodeling within the intracardiac nervous system. These shared pathways suggest that neuronal and myocardial fibrosis are interdependent processes governed by a common network of cytokine, immune, and mechanical cues, a concept explored in the subsequent sections of this review.

## 4. Neuroinflammatory and Fibrotic Remodeling of the Intrinsic Cardiac Nervous System

### 4.1. Metabolic and Cytokine-Induced Neurodegeneration

Evans and Li (2024) delineate how Type 2 Diabetes Mellitus provokes molecular and structural degeneration within the ICG, leading to impaired autonomic regulation [24]. They identify oxidative stress, advanced glycation end-products, and inflammatory cytokines such as IL-6 and TNF-α as principal mediators activating PI3K/AKT, MAPK/ERK, and RAGE signaling cascades, culminating in calcium-channel suppression and neuronal atrophy [24]. Similarly, another study provide anatomical evidence that chronic hyperglycemia in Goto–Kakizaki diabetic rats causes marked neuronal loss and ganglionic atrophy within the ICG, attributed to oxidative stress, advanced glycation, and microvascular insufficiency, mechanisms that disrupt parasympathetic signaling and heighten arrhythmic vulnerability [55]. Complementary findings by other researchers demonstrate that noradrenaline inhibits the non-quantal release of acetylcholine in the rat atrial myocardium through α_1_- and α_2_-adrenergic receptor activation, revealing a direct sympathetic–parasympathetic interplay within intracardiac parasympathetic terminals independent of ganglionic transmission [56]. This receptor-mediated cross-talk exemplifies interganglionic neuromodulation that may contribute to neurofibrotic or cardiac diabetic remodeling processes affecting autonomic balance [56]. In addition, Salomão and colleagues report that low-dose 5-fluorouracil (5-FU) induces oxidative stress, fibrosis, and inflammatory remodeling within the intrinsic cardiac nervous system of rats. Elevated IL-6, IL-1, and CINC-1 expression, together with diminished antioxidant activity, lead to neurodegeneration and fibroblast activation through JAK/STAT3, NF-κB, and TGF-β pathways, promoting myofibroblast differentiation, ECM accumulation, and neurofibrosis [57]. Moreover, an interesting study elucidates the ionic mechanisms of histamine-induced excitation in intracardiac ganglion neurons, showing that histamine activates H_1_ receptors to trigger Gq/PLC/IP_3_ signaling, calcium release, and TRPC channel opening, processes that enhance neuronal excitability and link immune signaling to cardiac autonomic modulation [58]. Finally, intrinsic cardiac neurons exhibit remarkable electrophysiological diversity [59], including a subset defined by calbindin-D28k expression [60]. These neurons display lower excitability, reduced N-type Ca^2+^ currents, and structural features reminiscent of diabetic and heart failure-induced remodeling, suggesting a role for vitamin D_3_-dependent calbindin signaling in autonomic regulation [60].

### 4.2. Neuroimmune Fibroblast-Mediated Remodeling

**Figure 3:**
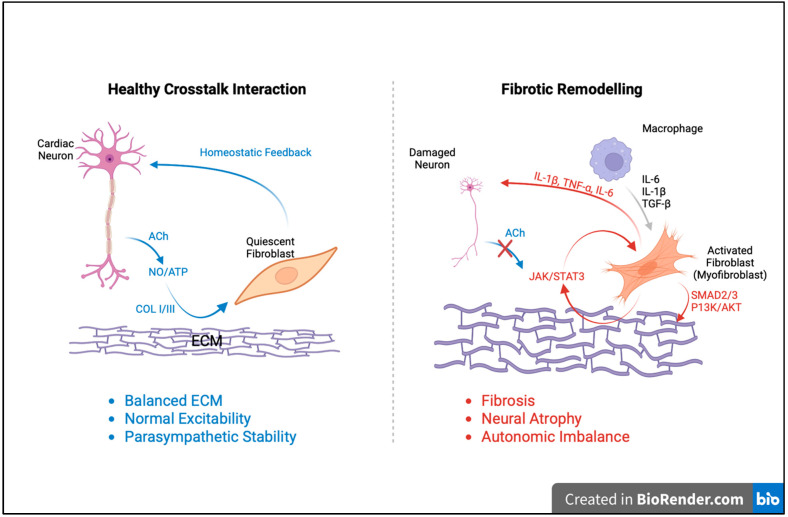
Neuro-fibrotic crosstalk regulating cardiac homeostasis and remodeling. In the healthy heart (**left**), cardiac neurons release ACh, NO, and ATP, maintaining quiescent fibroblasts and balanced ECM turnover through collagen I/III regulation. This bidirectional feedback supports normal excitability and parasympathetic stability. In contrast, during injury (**right**), neuronal damage and macrophage-derived cytokines (IL-1β, TNF-α, IL-6, TGF-β) activate fibroblasts via JAK/STAT3, SMAD2/3, and PI3K/AKT pathways, promoting fibrosis, neuronal atrophy, and autonomic imbalance. Created in Biorender. Gemayel, J.A. (2026) https://app.biorender.com/illustrations/canvas-beta/69047953250fcd558bc34ffa (accessed on 20 November 2025).

Several studies investigate MI-induced neurochemical remodeling in the porcine intrinsic cardiac ganglionated plexus and demonstrate that IL-6, TNF-α, and nitric oxide synthase activation drive neuroinflammation, glial activation, and fibrosis [61]. They document phenotypic shifts in neuronal markers, glial reactivity, and ECM deposition, evidencing neuron–glia–fibroblast coupling and implicating PI3K/AKT and MAPK/ERK-mediated pathways in neurofibrotic remodeling leading to autonomic imbalance. Abe and collaborators extend these findings by showing that IL-1-mediated inflammation in a mouse model of Kawasaki disease increases nerve growth factor and promotes sympathetic sprouting and immune infiltration into cardiac ganglia, whereas IL-1 receptor blockade prevents these effects, underscoring the role of cytokine-driven neuroinflammation and fibroblast activation in arrhythmogenic remodeling [62]. Other explore the brain-heart axis in subarachnoid hemorrhage, identifying cascading neurodegeneration across nodose, stellate, and intracardiac ganglia. Central nervous system injury initiates vagal ischemia, sympathetic hyperactivation, and fibroblast-associated neuronal degeneration, producing arrhythmias and cardiac injury. Their work introduces the concept of “Burned Neuro-Cardiac Web Syndrome,” emphasizing that PI3K/AKT- and MAPK/ERK-linked fibroblast responses extend neuroinflammatory damage from the brain to the heart through the vagal–intracardiac continuum [63]. Integrating these findings, Hanna and colleagues describe the intrinsic cardiac nervous system as a complex, hierarchical network capable of autonomous modulation, in which mixed cholinergic–adrenergic phenotypes and inflammatory signaling pathways, particularly MAPK/ERK and PI3K/AKT, mediate both adaptive and maladaptive remodeling [19]. Finally, Li and Bian identify fibroblast-derived IL-6 as a central autocrine mediator of CF after myocardial infarction. As depicted in Figure 3, the interaction between cardiac neurons and fibroblasts represents a crucial regulatory axis in both cardiac homeostasis and fibrotic remodeling. Under physiological conditions, neuronal signaling through acetylcholine (ACh), nitric oxide (NO), and ATP maintains fibroblasts in a quiescent state, ensuring balanced ECM turnover and preserving parasympathetic stability. In contrast, during disease or injury, neuronal damage and inflammatory cytokine release (including IL-1β, TNF-α, IL-6, and TGF-β) disrupt this communication, leading to fibroblast activation, myofibroblast differentiation, and excessive collagen deposition. These processes are mediated by JAK/STAT3, SMAD2/3, and PI3K/AKT signaling cascades and contribute to neural atrophy, autonomic imbalance, and progressive cardiac dysfunction. As well, Figure 3 illustrates how disruption of the cardioneuronal–fibroblast crosstalk shifts the myocardium from a state of homeostatic balance toward pathological fibrotic remodeling. Through fibroblast-specific knockout and molecular assays, they reveal an IL-6/STAT3/IL-11 cascade that drives myofibroblast activation and ECM expansion, situating fibroblast-secreted cytokine es as pivotal agents in PI3K/AKT and MAPK/ERK signaling networks and underscoring fibroblast-neuron crosstalk as a core mechanism of neurofibrotic remodeling [64]. As outlined in Table 1, fibrotic remodeling in the ICG arises from a network of neuro-glial-fibroblast-immune interactions mediated by signaling pathways such as IL-6/STAT3, NF-κB, PI3K/AKT, MAPK/ERK, and TGF-β. These interconnected mechanisms lead to fibroblast activation, glial reactivity, neuronal atrophy, and ECM accumulation, ultimately compromising autonomic regulation and promoting cardiac dysfunction.

### 4.3. Functional and Structural Remodeling of the Intrinsic Cardiac Nervous System

#### 4.3.1. Metabolic Stress-Mediated ICNS Remodeling (Diabetes)

Diabetes induces widespread structural and functional remodeling within the intrinsic cardiac nervous system, affecting both parasympathetic and sympathetic regulation [24,67]. In type 1 diabetes, significant neuronal atrophy and dystrophy occur within the pulmonary vein ganglia, leading to impaired parasympathetic control and a shift toward sympathetic predominance. This imbalance enhances susceptibility to atrial fibrillation and is accompanied by fibrosis and oxidative stress, emphasizing the multifactorial nature of diabetic remodeling and the potential value of targeting these pathways therapeutically. Metabolic dysregulation in type 2 diabetes further disrupts autonomic balance. Altered leptin signaling contributes to reduced cardiac vagal activity and impaired baroreflex sensitivity; leptin resistance and decreased UCP2 expression in cardiac vagal postganglionic neurons diminish parasympathetic tone and increase arrhythmic risk, highlighting both mitochondrial dysfunction and metabolic signaling as key therapeutic targets [68].

Type 2 diabetes-associated remodeling also extends to ICG neurons. Decreased neuronal excitability correlates with reduced N-type Ca^2+^ currents, as demonstrated in high-fat diet/low-dose streptozotocin models that reproduce hyperglycemia, hyperlipidemia, insulin resistance, and baroreflex impairment driven by calcium channel dysfunction [69]. Oxidative stress represents an additional mechanism governing diabetic neuronal remodeling. Hydrogen peroxide suppresses N-type Ca^2+^ channel expression in cardiac vagal postganglionic neurons, reducing their excitability and weakening vagal tone, whereas normalization of H_2_O_2_ levels via Ad.CAT gene therapy restores calcium currents, enhances vagal function, and reduces ventricular arrhythmogenesis [66]. Finally, decreased excitability of cardiac parasympathetic neurons in T2DM exacerbates post-myocardial infarction arrhythmias. Reductions in N-type Ca^2+^ currents and impaired nicotinic receptor signaling blunt vagal regulation while sympathetic output remains elevated, reinforcing the arrhythmogenic imbalance characteristic of diabetic autonomic dysfunction [70].

#### 4.3.2. Mechanical Load-Mediated ICNS Remodeling (Heart Failure)

Heart failure induces profound structural and functional remodeling within the intrinsic cardiac nervous system, where fibroblast-neuronal signaling, neurotrophic activation, inflammatory mediators, and ion-channel dysregulation converge to disrupt cardiac autonomic control. Cardiac fibroblasts, which become activated and proliferate during pathological remodeling, secrete cytokines, growth factors, and extracellular matrix components that reshape the neuronal microenvironment [71]. These fibroblast-derived signals contribute to electrical remodeling of intrinsic cardiac neurons, a hallmark of heart failure. Reduced N-type Ca^2+^ currents and diminished neuronal excitability have been demonstrated in postganglionic neurons [72], and complementary findings in atrioventricular neurons confirm suppression of N-type channel function and impaired autonomic transmission [73]. Cardiac neuronal hypertrophy within cardiac ganglia is a hallmark of heart failure. Elevated myocardial nerve growth factor (NGF) induces this hypertrophy and promotes heterogeneous sympathetic reinnervation after infarction, thereby enhancing electrophysiological dispersion and vagal withdrawal [74].

Fibroblast-mediated inflammation further influences neurochemical modulation within the ICNS. Bradykinin, produced in inflamed or fibrotic myocardium, directly excites autonomic neurons via B_2_ receptor activation, promoting Ca^2+^ influx and suppressing M-type K^+^ currents, thereby heightening neuronal depolarization and firing [75]. Ion-channel dysregulation further links fibroblast-driven inflammation to impaired autonomic regulation. Kv7/M currents, essential stabilizers of membrane potential in parasympathetic ganglia, become altered in conditions of chronic remodeling and inflammatory stress [76]. Heart failure–associated ICNS dysfunction also involves broader shifts in calcium-handling proteins, including altered Cav1.3 channel activity that affects brain–heart communication and ganglionic excitability [77]. These changes occur alongside structural and molecular remodeling of neuronal and fibroblast networks within intrinsic cardiac ganglia, which together contribute to disrupted autonomic integration and proarrhythmic behavior in heart failure [7]. Collectively, these findings highlight that autonomic dysfunction in heart failure arises not only from neuronal remodeling but from a tightly interwoven fibroblast-neuronal axis driving progressive electrical instability. Table 2 summarizes the disease-specific initiating factors, priority molecular pathways, ICNS remodeling characteristics, and potential therapeutic targets for major cardiovascular diseases. This horizontal comparison highlights both shared and distinct mechanisms of neurofibrotic remodeling, providing a framework for translational research and potential interventions.

## 5. Future Directions and Knowledge Gaps in Cardiac Neuron Research

Although significant progress has been made in understanding the molecular and functional aspects of the ICNS, substantial gaps remain regarding its role in cardiac pathology. Most studies have characterized the ICNS indirectly or in isolated models, leaving key questions about cellular heterogeneity, connectivity, and adaptive remodeling during disease unresolved. Future research should aim to fully elucidate intracardiac neurons and their interactions with cardiac fibroblasts, with a particular focus on the complex neuro-glial-fibroblast crosstalk that governs cardiac fibrosis and neuronal degeneration. Critical molecular pathways linking neuronal excitability, oxidative stress, and fibroblast activation within intracardiac ganglia remain incompletely defined. Advanced approaches such as single-cell transcriptomics, spatial proteomics, and high-resolution 3D imaging will be essential to construct a comprehensive cellular and molecular atlas of cardiac neurons and their supporting networks.

Translation of preclinical discoveries to human systems represents another major priority. The diversity, neurochemical complexity, and electrophysiological characteristics of human intracardiac neurons are still largely unexplored. Development of human cardiac tissue atlases, organoid co-culture platforms, and in vivo neuromodulatory imaging could provide insight into how neuronal remodeling contributes to arrhythmogenesis, heart failure, and fibrosis, and guide region-specific therapeutic interventions around pulmonary veins and atrioventricular junctions. Complementary analysis of human ganglionated plexus neurons has already revealed enhanced excitability, adrenergic shifts, and increased synaptic complexity in atrial fibrillation, implicating neuronal hyperexcitability in arrhythmogenic substrate formation.

Experimental strategies could include ICNS fibroblast-specific IL-6 knockout models to test whether inhibiting this pathway protects neurons and prevents fibrosis after myocardial infarction. Clinically, small trials could assess IL-6/STAT3-targeted therapies (e.g., tocilizumab) combined with neuromodulation (such as vagus nerve stimulation) to improve autonomic function and reduce neurofibrosis markers (e.g., serum IL-11). These approaches may target oxidative stress, cytokine signaling, calcium handling, and autonomic balance to preserve neuronal integrity and limit fibrotic transformation.

Several controversies and translational barriers warrant attention. First, whether ICNS neurons directly secrete profibrotic factors remains debated; neurons can release ATP to activate fibroblasts, but glial-mediated effects may dominate. Second, the causal relationship between neurofibrosis and myocardial fibrosis is unclear, with evidence supporting a bidirectional interplay. Translational challenges include the scattered and heterogeneous distribution of ganglia, multi-pathway crosstalk (e.g., IL-6/STAT3, TGF-β/SMAD, PI3K/AKT, MAPK/ERK), and species-specific differences in excitability and synaptic integration, which limit the applicability of preclinical models. Addressing these controversies and barriers is critical to guide effective ICNS-targeted therapies.

Ultimately, the future of cardiac neurobiology lies in integrating neural and myocardial physiology into a unified framework. Treating the ICNS as an active regulatory network rather than a passive relay may uncover therapeutic targets capable of restoring both electrical stability and structural integrity in the diseased heart, paving the way for neuroprotective and antifibrotic interventions that preserve cardiac autonomic function.

## 6. Conclusions

The ICNS represents a vital yet underappreciated regulatory network that integrates neural, glial, immune, and fibroblast signaling within the heart. Evidence now indicates that CF and neuronal remodeling are deeply interconnected processes, driven by shared molecular pathways involving oxidative stress, cytokine signaling, and fibroblast activation. Within the ICG, these mechanisms culminate in neural fibrosis, neuronal dysfunction, and impaired autonomic balance, key contributors to arrhythmogenesis and cardiac failure. In addition, the ICNS is an important control center in the heart, and its dysfunction contributes to both neural and heart muscle fibrosis. Key pathways, such as IL-6/STAT3, TGF-β/SMAD, and PI3K/AKT, play central roles in these processes and could be targeted for therapy. While studies in animals suggest that modulating the ICNS may improve heart function, applying this in humans is still difficult because of limited access to intracardiac neurons and incomplete knowledge of human ICNS. It is also unclear whether neurons directly release profibrotic factors and how much glial cells contribute, so more research is needed.

Finally, advances in molecular profiling, spatial mapping, and neuromodulatory technologies are rapidly transforming our understanding of this system. Future efforts should focus on defining and characterizing cell-specific interactions, clarifying the temporal sequence of neuronal and fibrotic remodeling, and developing therapies that preserve both neural and myocardial function. Recognizing the ICNS as an integrated component of cardiac pathophysiology offers not only new mechanistic insights but also transformative potential for therapeutic intervention across a broad spectrum of cardiovascular diseases.

## Figures and Tables

**Table 1:** Cellular and Molecular Pathways Driving Fibrotic Remodeling in the ICG.

Cellular Interaction	Key Molecular Mediators/Pathways	Experimental/ Disease Context	Evidence Level	Functional Consequence in Intracardiac Neurons	Representative References
Fibroblasts → Neurons	IL-6 → JAK/STAT3 → IL-11 axis; PI3K/AKT and MAPK/ERK signaling; RAGE activation by AGEs	Type 2 diabetes (Goto–Kakizaki rats), post-MI remodeling	Animal in vivo	neuronal atrophy, reduced excitability, autonomic imbalance, fibrosis	[24,55,64]
Immune Cells→ Neurons	IL-1β, TNF-α, IL-6; NF-κB and STAT3 signaling; cytokine-induced ROS	Kawasaki disease; 5-fluorouracil-induced neuroinflammation	Animal in vivo	Cytokine-mediated neuronal loss and sympathetic sprouting within ICG	[57,62]
Glia→ Neurons	IL-6/TNF-α; nitric oxide synthase; S100B, GFAP up-regulation; PI3K/AKT, MAPK/ERK	Porcine intrinsic cardiac plexus post-MI	Animal in vivo	Glial activation (gliosis), altered neurotransmission, fibrosis-associated neuronal dysfunction	[65]
Glia ↔ Fibroblasts	TGF-β, IL-6/STAT3, and NO signaling between activated glia and fibroblasts	Post-MI neuroinflammatory remodeling	Animal in vivo	Glial-driven fibroblast activation, ECM deposition, loss of ganglionic integrity	[63,65]
CNS ↔ ICG	PI3K/AKT, MAPK/ERK, IL-6 and TNF-α transmission along vagal–stellate pathways	Subarachnoid hemorrhage (“burned neuro-cardiac web”)	Animal in vivo	Secondary intracardiac neuroinflammation and fibroblast-associated degeneration	[63]
Oxidative Stress ↔ Neuronal Excitability	H_2_O_2_ → Ca^2+^ channel suppression; decreased antioxidant defense	T2DM ICG, post-MI arrhythmogenesis	Animal in vivo	Reduced parasympathetic tone, neuronal hypoexcitability, arrhythmogenic susceptibility	[24,66]
Fibroblast Cytokine Feedback ↔ ICG Neurons	IL-6/STAT3 autocrine signaling affecting neuronal PI3K/AKT pathways	Diabetic and post-MI neurofibrotic remodeling	Animal in vivo	Persistent cytokine-driven neuronal dysfunction and fibrosis	[24,64]
Neurons	Adrenergic shift, enhanced excitability, increased synaptic complexity	Human ganglionated plexus in atrial fibrillation	Human ex vivo tissue	Neuronal hyperexcitability contributing to arrhythmogenic substrate	[22]

Abbreviations: ICG = intracardiac ganglia; IL = interleukin; TNF = tumor necrosis factor; ROS = reactive oxygen species; RAGE = receptor for advanced glycation end-products; PI3K = phosphoinositide-3-kinase; MAPK = mitogen-activated protein kinase; ECM = extracellular matrix; MI = myocardial infarction; T2DM = type 2 diabetes mellitus.

**Table 2:** Disease-Specific Mechanisms of the Neurofibrotic Axis in the ICNS.

Disease Type	Initiating Factors	Priority Core Pathways	ICNS Remodeling Characteristics	Clinically Relevant Targets
Type 2 Diabetes	Oxidative stress + Advanced glycation	**PI3K/AKT > IL-6/STAT3** (main); AGE/RAGE, oxidative Ca^2+^ channel suppression (secondary)	Neuronal atrophy, reduced excitability, calcium channel inhibition	Antioxidants, IL-6 inhibitors
Myocardial Infarction	Inflammatory storm + Tissue necrosis	**TGF-β/SMAD > IL-27** (main); TNF-α/NF-κB, IL-1β, oxidative stress (secondary)	Gliosis, sympathetic sprouting, neuronal hypertrophy	Anti-TGF-β antibodies, IL-27 blockade
Heart Failure	Mechanical load + Neural remodeling	**MAPK/ERK > NGF** (main); PI3K/AKT, oxidative stress, Ca^2+^ channel dysfunction (secondary)	Neuronal hypertrophy, M-type K^+^ current abnormalities, autonomic imbalance	NGF neutralizing agents, ion-channel modulators

## Data Availability

No new data were created or analyzed in this study. Data sharing is not applicable to this article.
